# Practice Variations in the Use of Novel Oral Anticoagulants for Nonvalvular Atrial Fibrillation-Related Stroke among Stroke Neurologists in Saudi Arabia

**DOI:** 10.1155/2019/5373250

**Published:** 2019-08-21

**Authors:** Mohammed H. Alanazy, Taim Muayqil

**Affiliations:** Division of Neurology, Department of Internal Medicine, King Saud University Medical City and College of Medicine, King Saud University, Riyadh, Saudi Arabia

## Abstract

Clinical trials have demonstrated that novel oral anticoagulants (NOACs) are noninferior to warfarin in preventing nonvalvular atrial fibrillation- (nvAF-) related stroke and systemic embolism. However, in these trials, NOACs initiation was delayed for a variable period after stroke. Herein, we aimed to investigate the variability in early initiation of NOACs after nvAF-related stroke among stroke neurologists in Saudi Arabia. A standardized questionnaire was distributed electronically to all the stroke neurologists and fellows in Saudi Arabia. The questionnaire primarily focused on the timing of NOACs initiation after an nvAF-related stroke, according to stroke size (small, medium, and large) and location (anterior or posterior circulation). Thirty-six (85.7%) of the 42 stroke neurologists, who were contacted, participated in the survey. All participants would initiate NOACs in the first 3 days after a TIA; most of them initiate NOACs within 7 days after a small stroke, 4–14 days after a medium stroke, and ≥12 days after a large stroke, regardless of stroke location. Presence of a symptomatic intracranial hemorrhage further delayed initiation of NOACs. Additionally, 77.8% of the participants would bridge with antiplatelets before initiation of NOACs, and 55.6% would use a single antiplatelet agent. In conclusion, the practice of stroke neurologists is consistent with and supports the available evidence from observational studies on the time of initiation of NOACs. Our findings provide a guide for clinicians who manage nvAF-related stroke until more robust evidence from randomized controlled trials is available.

## 1. Introduction

Atrial fibrillation (AF) is one of the modifiable risk factors for stroke; it causes approximately one-fifth of ischemic strokes [1]. Anticoagulation with warfarin has been the hallmark of stroke prevention in cases of nonvalvular AF (nvAF) for decades. Since 2010, clinical trials on novel oral anticoagulants (NOACs: dabigatran, rivaroxaban, apixaban, and edoxaban) have shown that NOACs are at least noninferior to warfarin in preventing nvAF-related stroke and systemic embolism and have a lower rate of intracranial hemorrhage [2–5]. Both the American College of Cardiology/American Heart Association (ACA/AHA) and the European Society of Cardiology (ESC) guidelines recommend NOACs over warfarin for NOACs-eligible patients with nvAF [6, 7].

Because the NOACs clinical trials did not include patients within the first few weeks of ischemic stroke, the optimal timing of initiation of anticoagulation therapy remains uncertain; this is mostly due to concerns about hemorrhagic transformation of the index stroke. Paciaroni et al. reported, in a meta-analysis of randomized controlled trials, that using heparin within 48 hours of ischemic stroke resulted in a significantly increased risk of intracranial hemorrhage, without a reduction in the risk of recurrence of ischemic stroke [8]. This risk of hemorrhagic transformation with early anticoagulation should be weighed against the risks of recurrent stroke with delayed anticoagulation; the latter has been estimated to range from 0.5% to 1.3% per day in the first two weeks [8–11]. Since the introduction of NOACs into clinical practice, several observational studies have reported a low frequency of intracranial hemorrhage in patients who have received NOACs early after mild ischemic stroke [12]. One approach, based on expert consensus, suggests starting NOACs administration 1 day after transient ischemic attack (TIA), 3 days after mild stroke, 6 days after moderate stroke, and 12–14 days after large stroke [13]. However, while awaiting further guidance from randomized clinical trials, these decisions remain mostly individualized based on the risk/benefit assessment performed by the treating physician.

This study sought to investigate the variability in early initiation of NOACs after an nvAF-related stroke among stroke neurologists in Saudi Arabia.

## 2. Methods

The study was approved by the institutional review board at King Saud University Medical City, Riyadh, Saudi Arabia. The study included all adult stroke neurologists and fellows practicing in Saudi Arabia (*N* = 42). A questionnaire was developed by the authors to determine when stroke neurologists initiate anticoagulation with NOACs after an nvAF-related TIA or a small, medium, or large stroke (Supplementary Materials, [Supplementary-material supplementary-material-1]). We adopted the classifications of the locations and sizes of ischemic strokes used by Paciaroni et al. [14] when enquiring about the physician's decision to use anticoagulation. A small stroke was defined as infarct size ≤15 millimeter (mm) in the longest diameter in the anterior or posterior circulation. A medium supratentorial stroke was defined as infarct size ≤1/3^rd^ of the territory of the middle cerebral artery (MCA), anterior cerebral artery (ACA), or posterior cerebral artery (PCA), for example, cortical superficial branch of the MCA, ACA, or deep branch of the MCA. A large supratentorial stroke was defined as infarct involving the complete territory of the MCA, ACA, or PCA, or involving two cortical superficial branches of the MCA, one cortical and one deep branch of the MCA, or more than one arterial territory. Brainstem stroke was divided into small (≤15 mm) or large (>15 mm). Cerebellar stroke was divided into small (≤15 mm), medium (>15 mm and <½ of one cerebellar hemisphere), and large (>½ of one cerebellar hemisphere). The questionnaire also enquired about physicians' practice of bridging with antiplatelet administration and repeating brain imaging before initiation of NOACs, and the time of initiation of NOACs when there is asymptomatic or symptomatic hemorrhagic transformation. The questionnaire was independently reviewed by two stroke neurologists for content validity and readability.

A list of all stroke neurologists in Saudi Arabia was obtained from the Saudi Stroke Association. The questionnaire was electronically distributed via private messages between April 24 and 29, 2019. All participants signed an electronic informed consent form that was placed on the first page of the questionnaire.

Statistical analysis was conducted using the statistical software SPSS, version 23 (IBM, Armonk, NY). Data were reported as frequency and percentages. The Kruskal–Wallis test was used to evaluate the differences in time of NOACs initiation between different stroke locations of the same stroke size definition.

## 3. Results

A total of 42 stroke neurologists and fellows were contacted; 36 (85.7%) participated, of which 28 (77.8%) were men and 8 (22.2%) were women. The majority of participants (55.6%) were located in the Central province, 16.7% in the Western province, 22.2% in the Eastern province, 2.8% in the Northern province, and 2.8% in the Southern province. The majority of participants (44.4%) had 1–5 years of experience as a neurologist, 25.0% had 6–10 years of experience, 13.9% had 11–15 years of experience, 11.1% had 16–20 years of experience, and 5.6% had more than 20 years of experience.

**Figure 1:**
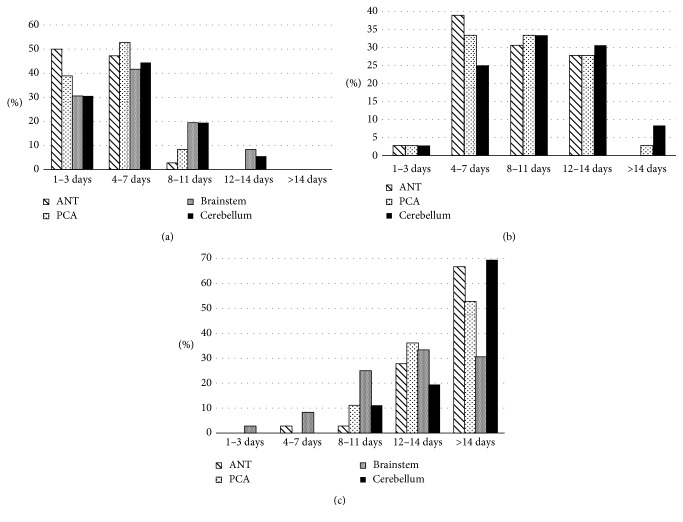
Participants' responses to the questions of when they would initiate NOACs after nvAF-related stroke, sorted according to stroke location and size. ANT, anterior circulation; PCA, posterior cerebral artery: (a) small stroke, (b) medium stroke, and (c) large stroke.

**Figure 2:**
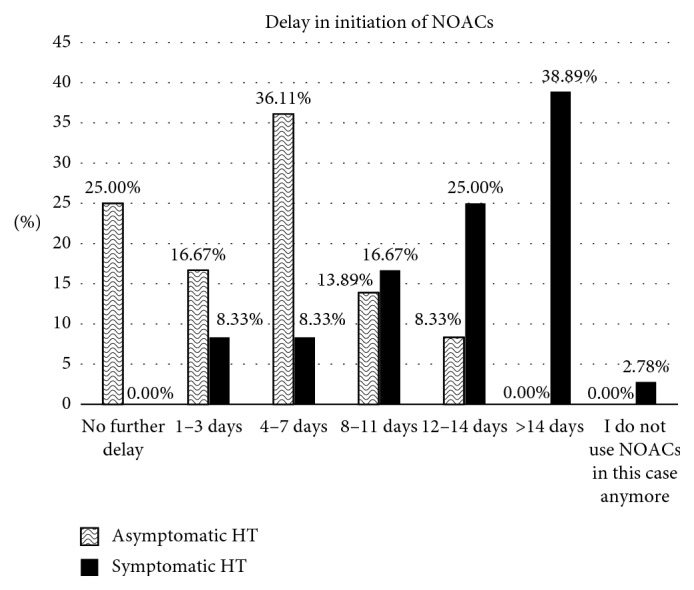
Participants' responses to the question of how many days they would further delay the initiation of NOACs if a pre-NOACs CT head shows hemorrhagic transformation (HT) of the index stroke.

The time of initiation of NOACs after nvAF-related stroke varied among participants according to stroke size and location, as shown in Table 1 and Figure 1. There were no significant differences in the timing of initiation of NOACs between the different stroke locations of the same size definition (the *P* values for small, medium, and large strokes were 0.39, 0.37, and 0.39, respectively). Regarding the question about repeating brain imaging before initiation of NOACs, 55.6% of the participants indicated that they repeat brain imaging regardless of the index stroke size, 25% would repeat brain imaging if the index stroke is medium or large, 14% would repeat brain imaging only if the index stroke is large, and 5.6% indicated that they do not repeat brain imaging. Participants' responses to the question of how many days they would further delay the initiation of NOACs if a pre-NOACs brain image shows symptomatic or asymptomatic hemorrhagic transformation (HT) of the index stroke are depicted in Figure 2.

Regarding the use of antiplatelet therapy as a bridge therapy before initiation of NOACs, 77.8% of the participants indicated that they use antiplatelets regardless of the index stroke size, 8.3% would use antiplatelets only when the index stroke is small or moderate, 2.8% would use antiplatelets only when the index stroke is small, and 11.1% do not use antiplatelet in this case scenario. Regarding the choice of antiplatelet agent, 55.6% would use a single antiplatelet agent, 11.1% would use dual antiplatelet agents, and 22.2% indicated that their choice is based on stroke size. Of all participants, 13.9% would use a single antiplatelet agent when the index stroke is large and dual antiplatelet agents when the index stroke is small.

## 4. Discussion

In this survey of stroke neurologists' practices of administering NOACs in nvAF-related stroke, we found that the practice was congruent with that seen in prospective observational studies globally, as well as the existing consensus guidelines. Correspondingly, the European Heart Rhythm Association and ESC allow commencing anticoagulants within 3 days for small strokes and TIAs, and after 12 days for large strokes [7, 13]. The ESC guidelines also recommend interrupting anticoagulant administration for 3–12 days in patients who suffer a moderate-to-severe ischemic stroke while on anticoagulants (level 2C). [7].

It is clear from the results (Table 1) that the confidence regarding initiating NOACs after small strokes with no complicating hemorrhage is high within the first two time groups, i.e., within 7 days, with greater confidence when encountering an anterior circulation stroke compared to strokes occurring in the posterior circulation. However, these differences were not statistically significant. This is interesting, given that recent studies have actually showed that hemorrhagic conversion is not common for posterior circulation strokes [15, 16]. When managing large brainstem strokes, respondents in the last two time periods were close, 33% and 30% for 12–14 and >14 days, respectively, in contrast to large strokes in the remaining regions wherein each more than 50% would wait >14 days. Even for mid-sized strokes, neurologists were observed to prefer waiting times that were mainly in between those of small- and large-sized strokes. Further, regarding the effect of stroke location (territory involved) on responses, the majority of participants start NOACs in the first week after a small stroke, 4–14 days after a medium-sized stroke, and ≥12 days after a large stroke regardless of their locations. When there was a hemorrhagic transformation of the index stroke, the answers were more consistent with a peak of responses in the 4- to 7-day period for asymptomatic hemorrhage and in >14 days for the symptomatic hemorrhage group. This is reasonable, considering that small hemorrhages are likely to be asymptomatic, and usually without a negative impact on functional outcome [15].

The ESC guidelines recommend considering aspirin for secondary stroke prevention in nvAF patients in whom a stroke occurs, until the initiation or resumption of NOACs therapy (level 2B) [7]. Similarly, most respondents in this study would opt to bridge with antiplatelets before initiating NOACs.

It appears that most of the neurologists surveyed in this study have carried over their anticoagulation decisions from previous guidelines on the topic. This is also expected, given the proven hemorrhagic risk with heparins [8]. Most responses indicated that stroke size was a major influencing factor in this study, which is not surprising, given its previously demonstrated association with hemorrhagic transformation [17]. The Early Recurrence and Cerebral Bleeding in Patients with Acute Ischemic Stroke and Atrial Fibrillation (RAF) study showed that initiating anticoagulation with any agent is reasonable within 4–14 days, and the risk of a recurrent ischemic event was stable for the first 2 weeks from the index event [14]. However, large lesions were associated with higher ischemic and hemorrhagic risks [14]. Interestingly, in one prospective study where 155 patients with an ischemic stroke or TIA were administered an NOAC, no intracranial hemorrhage occurred, whether NOAC was started before or after 7 days from the event [12]. In fact, recurrent ischemic events were more of a concern [12]. However, there were no specifics on stroke volume in that study [12]. Further prospective assessments found a low rate (2.4%) of hemorrhages occurring within 90 days of initiation of NOACs, and most were within 15 days [18]. In a single-center retrospective Japanese study, physicians appeared to be more comfortable in prescribing NOACs compared to prescribing warfarin, and their practice was similarly not associated with negative outcomes [19].

In this study, we chose not to include the National Institutes of Health Stroke Scale in our survey questions, as it may not correctly quantify deficits from the posterior circulation. The size definition we used in the anterior circulation and PCA was different than that used for the brainstem and cerebellum. A large stroke in the brainstem is smaller than a large MCA territory infarct. The definition of size appears to be relative to the infarcted structure and how much of the structure is functionally intact. This factor appears to be consistent when it comes to deciding about anticoagulation, rather than the actually measured size of the stroke.

Among the limitations to consider are that the responses here are opinions of neurologists for a hypothetical situation, and there are many other factors in the real world that need to be factored into the decision-making process. Many important variables that should be looked at in the future should also include the presence of microbleeds, cardiac thrombi, and patient age. Moreover, access to different NOACs varies among centers. Prospective observational studies of the actual practice of neurologists for when anticoagulation is started and the choice of drug to be used would be more representative. The small number of participants limited our ability to proceed with subgroup analyses; generally, no significant differences were found between the groups of participants based on their area of practice, years of experience, or sex (data not shown). Despite the small number, the response rate was more than adequate, and most respondents were in their first 5 years of practice, representing a new generation of stroke neurologists who are most likely to have gone through formal stroke fellowship programs. One ongoing trial should clarify some of the aforementioned issues; the New Oral Anticoagulants in Stroke Patients Long-Term Prevention of Recurrent Stroke in Patients with Atrial Fibrillation (NOACIST, NCT03826927) aims to provide real-world data on anticoagulation with both NOACs and vitamin K antagonists while also addressing early start of anticoagulation, and its effects on elderly and on those with previous intracranial hemorrhage. Other ongoing trials at the time of this writing include the OPTIMAS (NCT03759938), ELAN (NCT03148457), START (NCT03021928), and TIMING (NCT02961348) trials. All of these trials will look at the difference between early and late initiation of anticoagulation. The information from these trials would be most beneficial when the analysis of pooled results from individual participants is performed [12].

In summary, our goal here was mainly to determine the common practice among stroke neurologists towards a basic common scenario encountered and obtain a glimpse of a potential regional consensus. This will aid all physicians within the country who encounter these conditions, including internists, emergency physicians, rural physicians, or those involved in making regional consensus guidelines.

## Figures and Tables

**Table 1:** Time of initiation of NOACs after nvAF-related stroke based on stroke size and location.

Area	Stroke size	NOACs initiation after stroke (*n* = 36)				
		1–3 days (%)	4–7 days (%)	8–11 days (%)	12–14 days (%)	>14 days (%)
Any	TIA	100				

Anterior circulation	Small	50.0	47.2	2.8		
	Medium	2.8	38.9	30.6	27.8	
	Large		2.8	2.8	27.8	66.7

Posterior cerebral artery	Small	38.9	52.8	8.3		
	Medium	2.8	33.3	33.3	27.8	2.8
	Large			11.1	36.1	52.8

Cerebellum	Small	30.6	44.4	19.4	5.6	
	Medium	2.8	25.0	33.3	30.6	8.3
	Large			11.1	19.4	69.4

Brainstem	Small	30.6	41.7	19.4	8.3	
	Large	2.8	8.3	25.0	33.3	30.6

NOACs, novel oral anticoagulants; TIA, transient ischemic attack.

## Data Availability

The data used to support the findings of this study are included within the article.

## Supplementary Materials

Supplementary Materials The questionnaire used to collect data in this study.
