# Supplementary material for: Practice Variations in the Use of Novel Oral Anticoagulants for Nonvalvular Atrial Fibrillation-Related Stroke among Stroke Neurologists in Saudi Arabia
Source: Neurol Res Int. 2019 Aug 21;2019:5373250. doi: 10.1155/2019/5373250 (PMC6721244; doi:10.1155/2019/5373250)
Supplement: Supplementary Materials — The questionnaire used to collect data in this study. [file 5373250.f1.docx]

**Practice variations in novel oral anticoagulants use for non-valvular atrial fibrillation-associated stroke among stroke neurologists in Saudi Arabia**

1. Sex
   1. Male
   2. Female
2. Years of experience since obtaining Neurology board certificate.
   1. 1-5 years
   2. 6-10 years
   3. 11-15 years
   4. 16-20 years
   5. More than 20 years.
3. City of practice.
   1. Riyadh
   2. Jeddah
   3. Dammam
   4. Makkah
   5. Madinah
   6. Other (please specify)

________________

1. When do you initiate novel oral anticoagulants (NOAC) for eligible patients with a non-valvular atrial fibrillation-associated stroke or TIA (i.e No left atrial thrombus or hemorrhagic transformation) in each of the following case-scenarios:
   1. Acute TIA with no evidence of ischemic stroke on DWI?
      1. 1-3 days
      2. 4-7 days
      3. 8-11 days
      4. 12-14 days
      5. >14 days
   2. A small stroke (longest diameter ≤ 15 mm) in the **anterior circulation**?
      1. 1-3 days
      2. 4-7 days
      3. 8-11 days
      4. 12-14 days
      5. >14 days
   3. A **medium** stroke in the **anterior circulation** involving ≤1/3^rd^ of the territory of MCA, or ACA (e.g., involving a cortical superficial branch of the MCA, an MCA deep branch, or a cortical superficial branch of the ACA)?
      1. 1-3 days
      2. 4-7 days
      3. 8-11 days
      4. 12-14 days
      5. >14 days
   4. A **large** stroke in the **anterior** **circulation** (involving the complete territory of the MCA, or ACA, or involving 2 cortical superficial branches of the MCA, a cortical superficial branch of the MCA associated with a deep branch of the MCA, or involving >1 arterial territory [eg, both MCA and ACA territory])?
      1. 1-3 days
      2. 4-7 days
      3. 8-11 days
      4. 12-14 days
      5. >14 days
   5. A **small** stroke (longest diameter ≤ 15 mm) in the **PCA** territory?
      1. 1-3 days
      2. 4-7 days
      3. 8-11 days
      4. 12-14 days
      5. >14 days
   6. A **medium** stroke in the PCA territory i.e., involving ≤1/3^rd^ of the territory of **PCA**?
      1. 1-3 days
      2. 4-7 days
      3. 8-11 days
      4. 12-14 days
      5. >14 days
   7. A **large** stroke in the **PCA** territory (involving the complete territory of the PCA)?
      1. 1-3 days
      2. 4-7 days
      3. 8-11 days
      4. 12-14 days
      5. >14 days
   8. A **small** brainstem stroke (longest diameter ≤ 15 mm).
      1. 1-3 days
      2. 4-7 days
      3. 8-11 days
      4. 12-14 days
      5. >14 days
   9. A **large** brainstem stroke (longest diameter > 15 mm).
      1. 1-3 days
      2. 4-7 days
      3. 8-11 days
      4. 12-14 days
      5. >14 days
   10. A small **cerebellar** stroke (longest diameter ≤ 15 mm).
       1. 1-3 days
       2. 4-7 days
       3. 8-11 days
       4. 12-14 days
       5. >14 days
   11. A medium **cerebellar** stroke (longest diameter ≥15 mm and ≤ 1/2 of one cerebellar hemisphere).
       1. 1-3 days
       2. 4-7 days
       3. 8-11 days
       4. 12-14 days
       5. >14 days
   12. A large **cerebellar** stroke ( > 1/2 of one cerebellar hemisphere).
       1. 1-3 days
       2. 4-7 days
       3. 8-11 days
       4. 12-14 days
       5. >14 days
2. Do you use antiplatelets temporarily (either single or double) as a bridge therapy for a non-valvular atrial fibrillation-associated stroke if you have decided to delay initiation of NOAC? (**note: no atrial thrombus or hemorrhagic transformation)**
   1. No, not at all.
   2. Yes, I bridge with antiplatelet therapy only when the index stroke is small, but NOT if it was medium or large.
   3. Yes, I bridge with antiplatelet therapy only when the index stroke is small or medium, but NOT if it was large.
   4. Yes, I bridge with antiplatelet therapy regardless of the size of the index stroke.
3. With regards to the previous question, do you use single or double antiplatelet for bridging?
   1. No, I do not use antiplatelet therapy for bridging.
   2. A single antiplatelet therapy.
   3. Dual antiplatelet therapy.
   4. My choice of bridging with a single or dual antiplatelet therapy is based on the index stroke size (please explain)
4. Do you repeat brain imaging before initiating NOAC?
   1. No, not at all
   2. Yes, I request a pre-NOAC brain image only if the index stroke was large.
   3. Yes, I request a pre-NOAC brain image only if the index stroke was medium or large.
   4. Yes, I request a pre-NOAC brain image regardless of the index stroke size.
5. Do you further delay the initiation of NOAC if a pre-NOAC CT shows an **asymptomatic** hemorrhagic transformation (HT) of the index ischemic stroke? (note: asymptomatic = petechial or small ICH <10 mm with no worsening of NIHSS)
   1. No, I do not further delay initiation of NOAC in the case of asymptomatic HT.
   2. Yes, I delay initiation of NOAC for an additional 1-3 days after finding asymptomatic HT.
   3. Yes, I delay initiation of NOAC for an additional 4-7 days after finding asymptomatic HT.
   4. Yes, I delay initiation of NOAC for an additional 8-11 days after finding asymptomatic HT.
   5. Yes, I delay initiation of NOAC for an additional 12-14 days after finding asymptomatic HT.
   6. Yes. I delay initiation of NOAC for more than 14 days after finding asymptomatic HT.
6. When do you initiate NOAC if a pre-NOAC CT shows **symptomatic** hemorrhagic transformation (HT) of the index ischemic stroke? (symptomatic HT = ICH ≥ 10 mm or >30% of infarct volume with mass effect, and is associated with worsening of NIHSS)
   1. No, I do not further delay initiation of NOAC in the case of symptomatic HT.
   2. Yes, I delay initiation of NOAC for an additional 1-3 days after finding symptomatic HT.
   3. Yes, I delay initiation of NOAC for an additional 4-7 days after finding symptomatic HT.
   4. Yes, I delay initiation of NOAC for an additional 8-11 days after finding symptomatic HT.
   5. Yes, I delay initiation of NOAC for an additional 12-14 days after finding symptomatic HT.
   6. Yes, I delay initiation of NOAC for more than 14 days after finding symptomatic HT.
   7. No, I do not use NOAC in this case anymore.
